# Role of Simulated Training for Carotid Endarterectomy: A Systematic Review

**DOI:** 10.3400/avd.ra.22-00021

**Published:** 2022-12-25

**Authors:** Nadeem A. Siddiqui, Ammar Pirzada, Shoaib Badini, Fareed A. Shaikh

**Affiliations:** 1 Section of Vascular Surgery, Department of Surgery, Aga Khan University Hospital, Karachi, Pakistan

**Keywords:** simulated training, surgical education, surgical training, carotid artery surgery, carotid endarterectomy

## Abstract

Vascular surgery trainees often do not get to perform carotid endarterectomy (CEA) directly on the patients as it requires meticulous surgical technique and has a high risk of procedure-related complications. Hence, the role of simulation in training future vascular surgeons becomes essential. This review aims to assess the types and utility of simulators available for CEA. In this systematic review, all the studies performed on CEA simulation were included. The purpose of this review was to assess different types of simulators and their usefulness for CEA. We identified 122 articles, of which 10 were eligible for review. A variety of simulators, ranging from animal models, virtual reality simulators and commercially designed models with high fidelity options were used. Technical competence was the major domain assessed in the majority of the studies (n=8), whereas four studies evaluated anatomical and procedural knowledge. Blinding was done in five studies for assessment purposes. The majority of studies (n=9) found the simulation to be an effective tool for achieving technical competence. This review shows the potential usefulness of simulation in acquiring technical skills and procedural acumen for CEA. The available literature is unfortunately too diverse to have a common recommendation.

## Introduction

Traditionally, surgical trainees have been trained on patients in the operating room, which has led to concerns like patient safety and overall cost due to increased length of procedure.^1,2)^ Vascular surgery patients are generally more complex than other surgical specialties due to the nature of the disease.^3)^ This poses a significant challenge to learn specialised skills without compromising the outcomes. In addition, work hour restrictions,^4)^ reduced duration of residency,^5)^ medicolegal complaints^6)^ and an increasing acceptance of endovascular-first approach^7)^ have greatly affected surgical trainings in general and open procedural skills in particular.

Media reports like ‘Bad surgeons cannot be detected,’^8)^ reduced exposure to hands-on surgery and the subsequent lack of confidence in graduating trainees to perform independent procedures were some of the reasons behind using simulations for surgical trainings. Simulation can be simply defined as a method, which is deployed to produce an experience without going through the real event.^9)^ It allows for the safe practice of certain techniques and is becoming increasingly important in the shift towards improved education of trainees in surgery.^10)^ A recent systematic review by Lawaetz et al.^6)^ reported the benefit of simulated training in open vascular surgery; however, it included all open vascular procedures, whereas we aim to focus on Carotid Endarterectomy (CEA).

CEA is the standard open procedure aimed to prevent imminent stroke in selected patients.^11,12)^ In addition, guidelines for performing this complex procedure recommend that a vascular surgeon with high procedure volumes^13)^ and perioperative stroke risk less than the natural course of carotid artery disease is eligible.^14)^ This benchmark puts enormous pressure not only upon the novice surgical trainee but also on the supervising consultant, ultimately resulting in the consultant performing the main steps of every procedure.

Various simulation models, both bench top and bovine, have been described for the training of CEA with varying results.^3,5,15–21)^ Given the limited opportunity for trainees to perform CEA and the delicate nature of the procedure, we decided to conduct this systematic review to look at the different models available and assess their benefits. The main objective of this systematic review was to identify different types of simulators used for the training of CEA and to assess the usefulness of all such simulators considered for simulated training on CEA.

## Methods

This systematic review was conducted in line with the Preferred Reporting Items for Systematic Reviews and Meta-Analyses statement.^22)^ A systematic search for relevant studies was carried out, using search engines like Google Scholar, PubMed, Cochrane database, Medline and Scopus. Studies including observational to interventional trials, which reported the use of simulation in CEA were included. Exclusion criteria included simulation-based studies done solely on the vascular anastomosis, those involving animal research, in languages other than English and unpublished studies. Most of the innovations and advancements in the field of simulation in vascular surgery have been reported in the last two decades; hence, articles published after January 2000 were included, and the last date of the literature search was 31 August 2021.

### Search Strategy

To identify relevant studies, the search strategy was based on concepts of population, intervention and outcome. Population was identified as trainees of vascular surgery programmes. The search terms used to look for population were ‘vascular surgery trainees’ OR ‘fellows of vascular surgery’ OR ‘residents of vascular surgery’ OR ‘vascular physicians’ OR ‘vascular surgeons’ AND ‘carotid endarterectomy’ OR ‘open surgery for carotid artery stenosis’ OR ‘carotid artery surgery.’ Intervention of interest was simulation models used for CEA. For that, we used ‘simulation models’ OR ‘simulation tools’ OR ‘simulation training’ OR ‘simulation in vascular surgery’ as search terms. Outcome was the effectiveness of different types of simulators used for CEA. For such outcome, search terms used were ‘effectiveness’ OR ‘efficacy’ OR ‘usefulness’ OR ‘Impact’ OR ‘benefits’ OR ‘role’ AND ‘simulator types’ OR ‘simulation in carotid endarterectomy.’

With the use of the relevant search for the identified terms, two investigators individually searched and reviewed the literature. In the case of any disagreement between the two reviewers, a third reviewer was included. For inclusion of the studies, an initial screening was done by reading the title of the study and identifying duplicates. Final inclusion was judged after reviewing the abstracts and evaluating the manuscript. To avoid missing any relevant studies, references to the included studies were also searched separately. The quality of included studies was analysed using the National Institutes of Health (NIH) scoring system,^23)^ which is a validated tool used to assess and rate the quality of research studies used for the purpose of systematic reviews and meta-analysis. For assessment of study quality, the Agency for Healthcare Research and Quality (AHRQ) grading system, which is a nine-point grading system on the basis of which studies are classified as poor, fair, good or excellent based on the allocated numbers during review, was also used in the study.^24)^

### Data Extraction

Data were collected on a predefined template that had information about publication year, journal name, first author name, country of origin, type of study, type of simulator, number of participants, level of training of participants, mode of assessment and overall outcomes of the paper.

### Operational definition

#### 1. Carotid Endarterectomy (CEA)

An open vascular surgical procedure performed to clear the carotid bifurcation of atherosclerotic plaque; hence, it prevents imminent stroke in a selected group of patients.^25)^

#### 2. Simulation

A situation in which a particular set of conditions is created artificially to study or experience something that is possible in real life or a generic term that refers to the artificial representation of a real-world process to achieve educational goals via experimental learning.^26)^

#### 3. Simulator

A device that enables the operator to reproduce or represent under test conditions phenomena likely to occur in actual performance.^26)^

## Results

**Figure 1:**
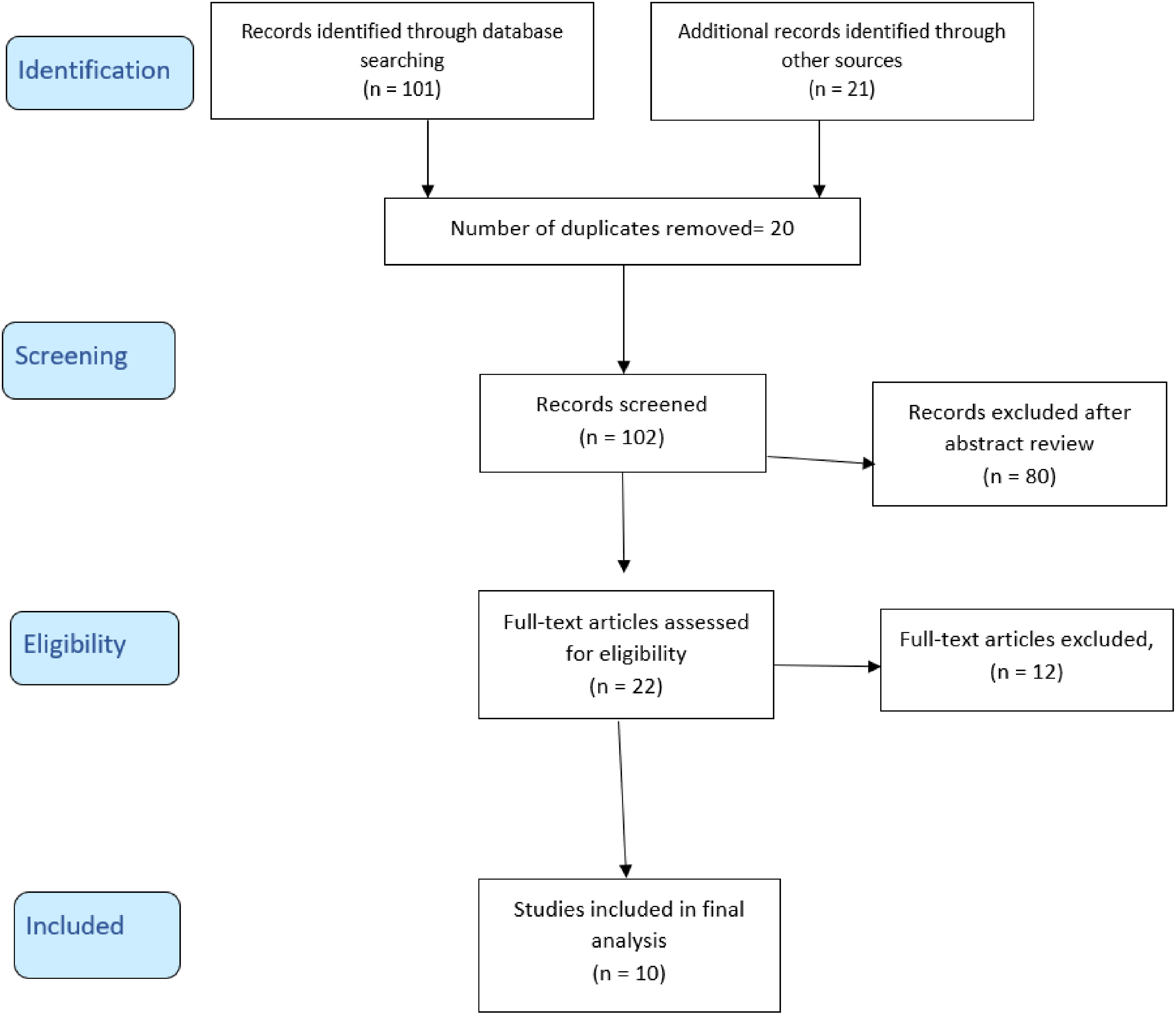
Fig. 1 Flow chart of preferred reporting items for systematic reviews and meta-analyses showing the study selection criteria for systematic review.

A total of 122 studies were retrieved after applying the search strategy, of which 10 met the inclusion criteria as shown in **Fig. 1**. Most of the studies were published after 2010. There were no study participants in Fletcher’s study.^17)^ In the remaining nine studies, the mean number of participants was 38 (range 6–165), as summarised in **Table 1**.

**Table 1:** Table 1 Summary of the basic characteristics of studies included in this systematic review

Author	Journal	Year of publication	Number of participants
Belykh^15)^	Neurosurgery	2015	06
Fletcher^17)^	Journal of Vascular Surgery Cases and Innovative Techniques	2017	Nil
Santangelo^16)^	Operative Neurosurgery	2018	Total-10 Novice-5 Expert-5
Sigounas^18)^	Simulation in Healthcare	2012	07
Black^3)^	British Journal of Surgery	2007	41
Black^19)^	British Journal of Surgery	2010	30
Duschek^5)^	Journal of Vascular Surgery	2013	10
Kim^21)^	Journal of Surgical Education	2016	Total-165 Resident-133 Consultant-32
Fletcher^20)^	Journal of Surgical Education	2020	17
Robinson^27)^	Journal of Vascular Surgery	2017	Total-58 Junior resident-33 Senior resident-25

One of these studies describes the development of the simulation model and did not involve any subjects for model validation.^17)^ Eight out of 10 focused on technical skills as their major outcome.^3,5,16,18–21,27)^ Two studies focused on anatomical knowledge and non-technical skills such as ‘crisis management’ in addition to procedural skills.^16,19)^ In one of the studies, knowledge of procedure and confidence level were both assessed during simulation-based learning.^20)^

**Figure 2:**
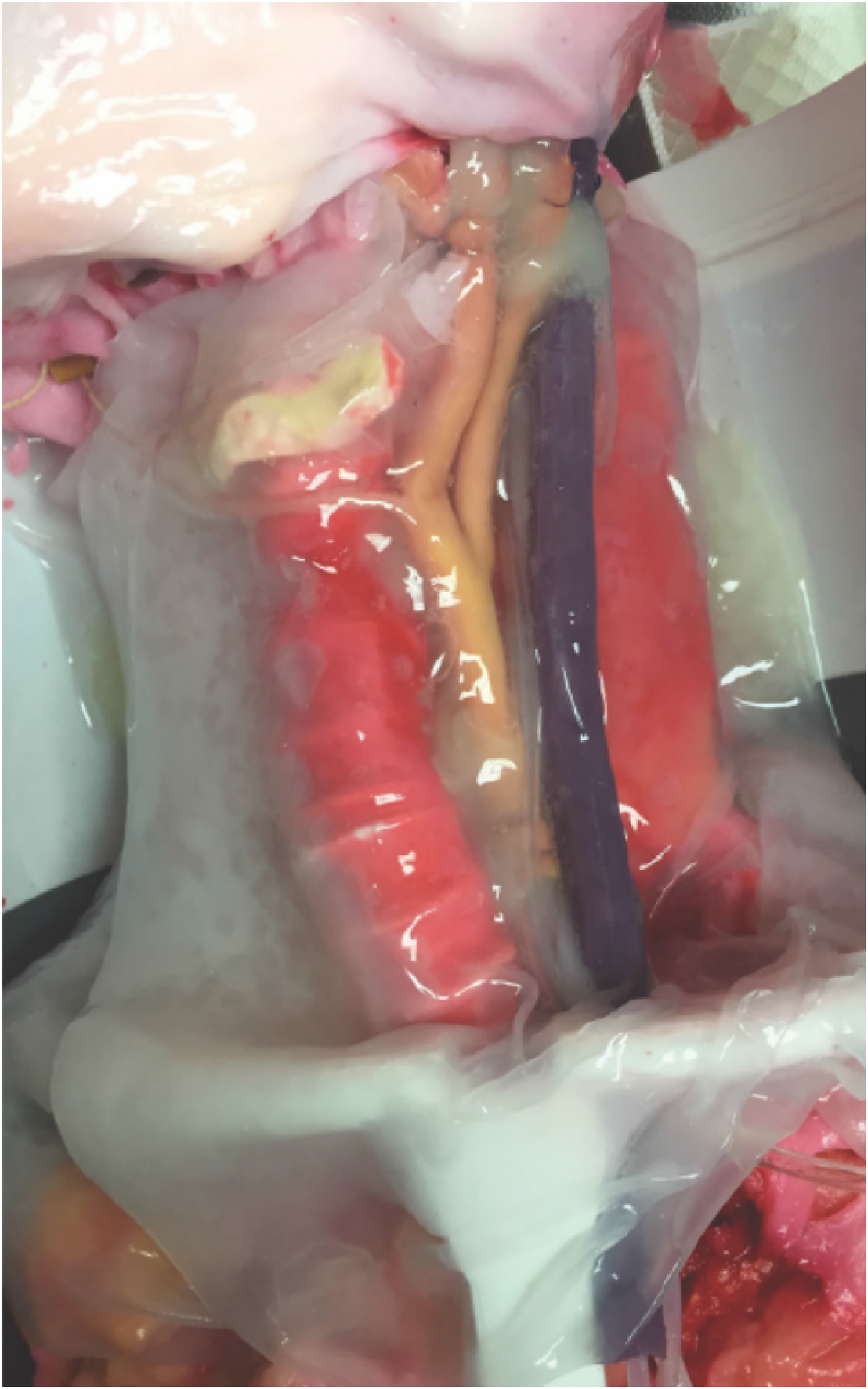
Fig. 2 Assembly of the simulated neck at the level of the carotid sheath with the visible carotid artery, jugular vein and vagus nerve.

**Figure 3:**
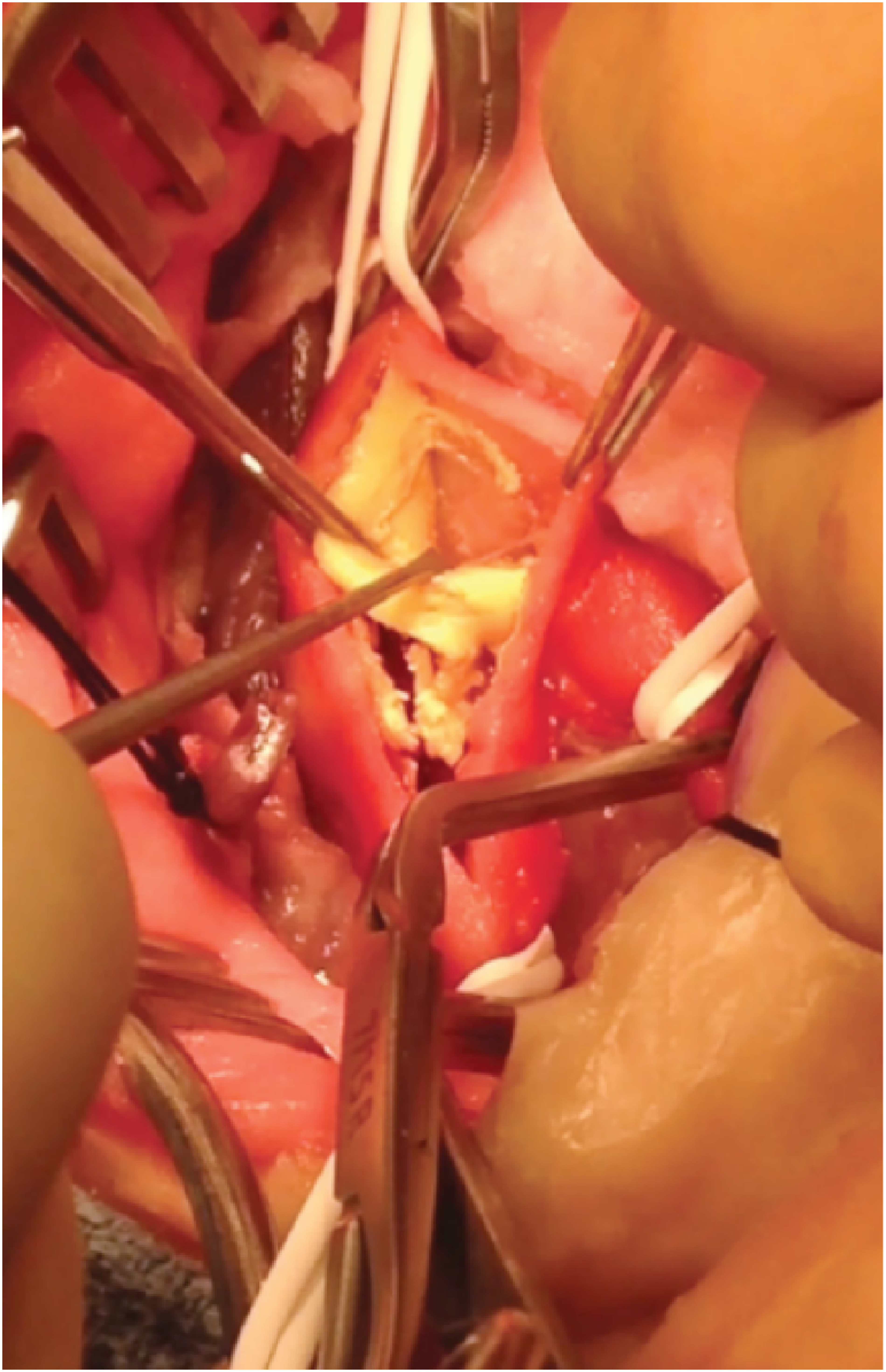
Fig. 3 Dissection of the plaque in the correct subintimal plane during cross-clamping.

The types of simulators used to teach CEA varied in different studies (**Table 2**). Five studies reported using plastic models, of which only two used pulsatile models.^5,17)^ Bovine placental model was used in one study,^15)^ whereas 3D printed whole task CEA simulator (made of polyvinyl alcohol) was used in another study^16)^ (**Figs. 2** and **3** added after permission for reprint from the corresponding author of publication). Moderate to high fidelity models were used in three studies,^17,18,27)^ whereas another three studies used cadaveric and cryopreserved models.^20,21,27)^ Assessment of learning was done in eight studies, whereas two studies did not mention any assessment strategy.^15,17)^ Modified Objective Structured Assessment of Technical Skills was the most commonly used assessment tool in four studies.^3,16,18,19)^ Imperial College Evaluation of Procedure-Specific Skill (ICEPS) rating scale,^3,19)^ Modified global rating scale (GRS)^5,21)^ and non-validated self-developed checklists^20,27)^ were among the other assessment tools. All the studies video recorded the learners’ performances, which were later on assessed by experts according to the above-mentioned rating scales. Four studies used a quasi-experimental design with a pre- and post-test, whereas the assessors were blinded to the participants.

**Table 2:** Table 2 Summary of the types of simulators, assessment tools and outcomes in different studies on simulation on CEA

Author	Type of simulation model	Domain assessed	Assessment done	Assessment tool	Outcome	NIH score
Belykh^15)^	Bovine placenta based model	Not reported	No	Nil	Beneficial	5
Fletcher^17)^	Pulsatile tissue based simulator	Technical skills	No	Nil	Probably	4
Santangelo^16)^	Whole task 3D printed PVA gel based model	Anatomical knowledge, technical skills	Yes	OSATS	Beneficial	8
Sigounas^18)^	Virtual reality simulator	Technical skills	Yes (blinded, pre and post)	Modifies OSATS, Fann’s rating score	Beneficial	7
Black^3)^	Synthetic latex CEA model	Knowledge/ technical skills	Yes (blinded)	OSATS, ICEPS	Beneficial	8
Black^19)^	Bench top model	Technical and non-technical skills	Yes (blinded)	OSATS, ICEPS, NOTECHS	Not known	8
Duschek^5)^	Pulsatile plastic bench model	Technical skills	Yes (blinded, Pre and post)	GRS, Task-specific checklist	Beneficial	8
Kim^21)^	Cadaveric model	Technical skills	Yes (blinded)	Modified GRS	Beneficial	8
Fletcher^20)^	Cryopreserved bench top moderate fidelity model	Procedure-specific knowledge, confidence and comfort	Yes (pre and post)	Numerical Likert scale	Beneficial	8
Robinson^27)^	Cadaveric model/ high fidelity Pontresina model	Procedural knowledge/technical skills	Yes (self pre and post)	Self-evaluation with checklist	Beneficial	8

CEA: Carotid Endarterectomy; NIH: National Institute of Health; PVA: polyvinyl alcohol; OSATS: Objective Structured Assessment of Technical Skills; ICEPS: Imperial College Evaluation of Procedure-Specific Skills; NOTECHS: Non-technical skills; GRS: Modified global rating scale

Eight out of 10 studies concluded that simulation was beneficial for teaching the procedure of interest. However, the study by Black did not conclude anything,^19)^ whereas in one by Fletcher et al., the efficacy of the simulator used was found to be inconclusive.^17)^

The mean NIH score^23)^ of all included studies was 7.2 (ranging from 5 to 8). According to AHRQ grading,^24)^ two studies were found to be of ‘poor’ quality,^15,17)^ whereas the rest of the studies were scored as ‘fair’ or ‘good.’

## Discussion

This systematic review has identified the paucity of literature on the role of simulation for CEA. Simulated training for CEA was found to be beneficial to teach and refine the technical skills of the trainees as well as the young vascular surgeons. Different models both pulsatile and non-pulsatile, cadaveric as well as synthetic, have been proposed with some advantages and disadvantages.

Simulation-based training gained importance since it provides an opportunity to improve not only the surgical skills but also the decision-making and behavioural response of a trainee surgeon in a critical situation within a risk-free environment.^28)^ This is essential for a procedure like CEA, where one of the major risks is stroke with a very limited margin of error. Most of the studies have focused on skill acquisition or procedural knowledge, but the final outcome of CEA also depends on non-technical aspects. Soft skills like communication, professionalism, team management, crisis handling and confidence are some important factors that play crucial roles in the final outcome of surgery. Two of the studies in this review^20,21)^ have addressed simulation on non-technical aspects like comfort and confidence of surgeons.

Various models have been described for simulation-based training on CEA. Among those, ‘Bovine Placenta Model’ was described by Belykh et al.^15)^ According to the authors, this model was found to be extremely useful since the artery in bovine placental tissue resembles the carotid artery in terms of anatomy and is generally available and inexpensive. Nevertheless, the major limitations of this model include the location of the dairy nearby hospital to deliver fresh specimens, infection control measures, inability to simulate it with the diseased vessels and lack of surrounding anatomical structures like internal jugular vein, vagus and hypoglossal nerves.^15)^ In certain geographical locations, the availability of bovine placenta may not be an issue due to the abundance of cattle farms. However, storage, delivery and biological hazard and infection control are potential concerns. ‘Cryopreserved pulsatile benchtop model’ described by Fletcher et al. is a portable, cost-effective and relatively easy-to-make model.^17)^ It requires cryopreserved vessels as the main component, which can affect its widespread availability since the process for cryopreservation does not exist in many countries.

Sigounas et al. used a plastic benchtop model called ‘Virtual Reality Simulator’.^18)^ They used three models for CEA, aortic aneurysm repair and femoropopliteal bypass. Compared to those in a wet laboratory, the main qualities of these simulators described by the authors were real-time simulation in terms of exposure, application of retractors, identification of structures and vascular anastomosis with high fidelity and minimum manpower required. Although it sounds attractive, the cost of these models and the simulation laboratory is a major challenge for implementation in low-income middle-class countries.

The positive impact of simulated training on the technical skills for CEA was confirmed by the majority of these studies by means of objective assessment. Video-recording the skills and rating them later helped reduce the risk of assessor performer bias as well as cope with the limited number of assessors available. Most of these assessment tools and checklists are generic in nature or non-validated/self-developed tools. This heterogeneity and the lack of standardised use of assessment tools among different studies limit our ability to conclude the true effectiveness of simulation in carotid surgery.

This review has some limitations including the relatively low number of participants in included studies. Included participants have diverse and varying exposure to vascular procedures, which can ultimately affect the effectiveness of simulated training on overall outcomes. Another limitation is the low number of available studies with significant heterogeneity in their methodology and assessment, which compromises our ability to truly gauge the educational impact of simulated training on the outcomes after carotid endarterectomy. Similarly, how much simulated training will translate into actual clinical practice with better outcomes remains not known. Despite all this, this is one of the first systematic reviews to comprehensively assess the status of focused simulation in carotid surgery and points towards the need for planning better studies and assessment tools for carotid artery surgeries.

## Conclusion

This review shows that the available literature for simulated training on carotid endarterectomy is useful in acquiring technical skills and procedural knowledge; however, the evidence is too diverse to conclude the superiority of one model over another. This demands better studies with a structured assessment to validate the simulators.
